# ^68^Ga-PSMA and ^68^Ga-DOTA-RM2 PET/MRI in Recurrent Prostate Cancer: Diagnostic Performance and Association with Clinical and Histopathological Data

**DOI:** 10.3390/cancers14020334

**Published:** 2022-01-11

**Authors:** Paola Mapelli, Samuele Ghezzo, Ana Maria Samanes Gajate, Erik Preza, Anna Palmisano, Vito Cucchiara, Giorgio Brembilla, Carolina Bezzi, Riccardo Rigamonti, Patrizia Magnani, Elisa Toninelli, Valentino Bettinardi, Nazareno Suardi, Luigi Gianolli, Paola Scifo, Alberto Briganti, Francesco De Cobelli, Antonio Esposito, Maria Picchio

**Affiliations:** 1 Vita-Salute San Raffaele University, Via Olgettina 60, 20132 Milan, Italy; mapelli.paola@hsr.it (P.M.); ghezzo.samuele@hsr.it (S.G.); palmisano.anna@hsr.it (A.P.); cucchiara.vito@hsr.it (V.C.); brembilla.giorgio@hsr.it (G.B.); bezzi.carolina@hsr.it (C.B.); toninelli.elisa@hsr.it (E.T.); briganti.alberto@hsr.it (A.B.); decobelli.francesco@hsr.it (F.D.C.); esposito.antonio@hsr.it (A.E.); 2 Nuclear Medicine Department, IRCCS San Raffaele Scientific Institute, Via Olgettina 60, 20132 Milan, Italy; samanesgajate.anamaria@hsr.it (A.M.S.G.); preza.erik@hsr.it (E.P.); rigamonti.riccardo@hsr.it (R.R.); magnani.patrizia@hsr.it (P.M.); bettinardi.valentino@hsr.it (V.B.); gianolli.luigi@hsr.it (L.G.); scifo.paola@hsr.it (P.S.); 3 Department of Radiology, IRCCS San Raffaele Scientific Institute, Via Olgettina 60, 20132 Milan, Italy; 4 Department of Urology, Division of Experimental Oncology, URI, Urological Research Institute, IRCCS San Raffaele Scientific Institute, Via Olgettina 60, 20132 Milan, Italy; 5 IRCCS Ospedale Policlinico San Martino, University of Genoa, Via Benzi 10, 16132 Genoa, Italy; nazarenoroberto.suardi@unige.it

**Keywords:** prostate cancer, recurrence, PSMA, RM2, PET/MRI

## Abstract

**Simple Summary:**

Prostate cancer (PCa) relapse occurs in up to 50% of patients after radical treatment. Once PCa recurrence is detected, a precise identification of the number and sites of recurrence is necessary to tailor the treatment on the patient’s needs. Positron emission tomography (PET) plays a pivotal role in this clinical setting and new radiotracers have been developed to improve its performance. While ^68^Ga-PSMA is a well-established radiotracer for PCa recurrence detection, ^68^Ga-DOTA-RM2 is a recently proposed tracer that targets the gastrin-releasing peptide receptors that are overexpressed in prostate cancer. In this work, the performance of ^68^Ga-PSMA and ^68^Ga-DOTA-RM2 PET/MRI in identifying recurrent disease were compared on the same cohort, using the same study protocol, as this is the only way to assess whether one outperforms the other and therefore should be preferred in clinical practice. Furthermore, the association between PET findings and clinical and histopathological characteristics was investigated to find potential biomarkers.

**Abstract:**

The aim of the present study is to investigate and compare the performances of ^68^Ga-PSMA and ^68^Ga-DOTA-RM2 PET/MRI in identifying recurrent prostate cancer (PCa) after primary treatment and to explore the association of dual-tracer PET findings with clinical and histopathological characteristics. Thirty-five patients with biochemical relapse (BCR) of PCa underwent ^68^Ga PSMA PET/MRI for restaging purpose, with 31/35 also undergoing ^68^Ga-DOTA-RM2 PET/MRI scan within 16 days (mean: 3 days, range: 2–16 days). Qualitative and quantitative image analysis has been performed by comparing ^68^Ga-PSMA and ^68^Ga-DOTA-RM2 PET/MRI findings both on a patient and lesion basis. Clinical and instrumental follow-up was used to validate PET findings. Fisher’s exact test and Mann-Whitney U test were used to investigate the association between dual-tracer PET findings, clinical and histopathological data. *p*-value significance was defined below the 0.05 level. Patients’ mean age was 70 years (range: 49–84) and mean PSA at time of PET/MR scans was 1.88 ng/mL (range: 0.21–14.4). A higher detection rate was observed for ^68^Ga-PSMA PET/MRI, with more lesions being detected compared to ^68^Ga-DOTA-RM2 PET/MRI (26/35 patients, 95 lesions vs. 15/31 patients, 41 lesions; *p* = 0.016 and 0.002). ^68^Ga-PSMA and ^68^Ga-DOTA-RM2 PET/MRI findings were discordant in 11/31 patients; among these, 10 were ^68^Ga-PSMA positive (9/10 confirmed as true positive and 1/10 as false positive by follow-up examination). Patients with higher levels of PSA and shorter PSA doubling time (DT) presented more lesions on ^68^Ga-PSMA PET/MRI (*p* = 0.006 and 0.044), while no association was found between PET findings and Gleason score. ^68^Ga-PSMA has a higher detection rate than ^68^Ga-DOTA-RM2 in detecting PCa recurrence. The number of ^68^Ga-PSMA PET positive lesions is associated with higher levels of PSA and shorter PSA DT, thus representing potential prognostic factors.

## 1. Introduction

Prostate cancer (PCa) relapse affects up to 50% of patients treated with radical prostatectomy (RP) or external-beam radiotherapy (EBRT) as primary treatment for clinically localized disease [1].

When biochemical recurrence (BCR) is detected, an accurate identification of location and extent of cancer recurrence is mandatory in order to address patients to directed therapies with prolonged intervals of cancer-free survival, thus avoiding systemic treatments, including androgen deprivation therapy [2].

Prostate Specific Antigen (PSA) is the commonly used circulating biomarkers identifying BCR; however, it is unable to provide information regarding the exact location of disease recurrence.

Of note, patients with low values of PSA might benefit the most by receiving early local therapies with curative intent; however, conventional imaging techniques have limited sensitivity in detecting tumour recurrences at PSA values lower than 1 ng/mL [1]. On the other hand, patients presenting metastatic disease should be candidate to systemic therapies. Therefore, the distinction between local or systemic disease is of utmost importance to plan the most appropriate treatment [3]. In this scenario, imaging plays a fundamental role in the identification of local and/or distant metastases.

In the last decade, Prostate Specific Membrane Antigen (PSMA), a transmembrane protein with a significantly increased expression in PCa cells, has gained particular attention as a radiotracer for positron emission tomography (PET) imaging in PCa, in view of its better sensitivity and specificity in detecting of metastatic disease, compared to conventional imaging, also at low PSA values [4,5,6].

Additionally, gastrin releasing peptide receptors (GRPR) are G-protein coupled receptors overexpressed in prostate tumours both at the mRNA and the protein level [7]. For this reason, GRPR analogues have been recently identified as alterative targets for molecular imaging in prostate cancer [8]. So far, among the available radiotracers targeting GRPR, ^68^Ga-DOTA-RM2 has been the most investigated [9,10,11,12].

Very few studies have been reported on the combined use of both ^68^Ga-PSMA and ^68^Ga-DOTA-RM2 in prostate cancer setting, both in the staging [12,13] and restaging [10,14] phase of the disease, with the majority of them using PET/computed tomography (CT) scanners for imaging acquisition [10,13,14].

The recent introduction of fully hybrid PET/Magnetic Resonance Imaging (MRI) paves the way to a completely innovative imaging approach for PCa recurrence. The simultaneous acquisition of PET and multiparametric MR (mp-MR) provides metabolic, structural, and functional imaging information regarding PCa status in a whole-body single session examination, with better soft tissue contrast and reduced radiation exposure in comparison to PET/CT [15,16]. However, very limited data are currently available on its role in this specific clinical setting. In particular, studies using both ^68^Ga-PSMA and ^68^Ga-DOTA-RM2 with PET/MRI are still anecdotal [10,12,14]

Therefore, the aim of our study was to compare the performances of ^68^Ga-PSMA and ^68^Ga-DOTA-RM2 PET/MRI in the identification of recurrent PCa after primary treatment. Furthermore, we also investigated the association between ^68^Ga-PSMA and ^68^Ga-DOTA-RM2 PET/MRI positive findings and clinical, as well as histopathological characteristics.

## 2. Materials and Methods

### 2.1. Patients

In this prospective clinical study, 35 consecutive patients with biochemically recurrent PCa were enrolled from June 2020 to March 2021 at IRCCS San Raffaele Scientific Institute.

Inclusion criteria were age greater than 18 years at the time of PET/MRI scan, previous radical treatment for PCa and biochemical failure, defined as two consecutive PSA measurements ≥ 0.2 ng/mL after RP or an increase by ≥2 ng/mL above the nadir PSA for patients that underwent RT [17].

Exclusion criteria were medical condition possibly interfering and significantly affecting study compliance and contraindications to undergo MRI scan (i.e., severe claustrophobia).

All recruited patients underwent ^68^Ga-PSMA PET/MRI and 31/35 also ^68^Ga-DOTA-RM2 PET/MRI in two different scan sessions, with at least 48 h interval.

PSA level at different time points and Gleason Score (GS) after RP, whenever available, were collected for all patients.

This study was approved by the Institutional Ethics Committee of IRCCS San Raffaele Scientific Institute (EudraCT 2018-001036-21), and all patients gave written informed consent to participate to the study.

The data presented in the present manuscript are part of a single-centre prospective study still ongoing at IRCCS San Raffaele Scientific Institute (Project GR-2016-02363991).

### 2.2. ^68^Ga-PSMA PET/MRI Acquisition Protocol

^68^Ga-PSMA was synthetized according to the procedure previously described. [12]

Fasting condition was requested on the day of ^68^Ga-PSMA PET/MRI scan. Images were acquired on a fully hybrid 3 Tesla PET/MRI system (SIGNA PET/MRI; General Electric Healthcare, Waukesha, WI, USA) from the skull base to mid-thigh in the following examined patients.

The ^68^Ga-PSMA PET/MRI scan started approximately 60 min (Mean ± SD, 63 ± 17 min) after injection of 129–288 MBq (mean ± SD, 170.56 ± 36.06 MBq) of ^68^Ga-PSMA [10,14].

The ^68^Ga-PSMA PET/MR examination protocol included a high statistic (HS) scan of the pelvis (20 min), covering a single bed position, that was simultaneously acquired with the following MRI sequences.

- an axial T2 weighted sequence with large field of view (FOV): FSE, TR = 10,235 ms; TE = 99.7 ms, FOV = 32 × 32 cm^2^; voxel size = 0.9 × 0.9 × 5 mm^3^,
- an axial T2 weighted sequence with small FOV: PROPELLER, TR = 9578 ms, TE = 151 ms, FOV = 18 × 18 cm^2^, voxel size = 0.6 × 0.6 × 3 mm^3^,
- a sagittal T2 weighted sequence with small FOV: PROPELLER, TR = 9578 ms, TE = 151 ms, FOV = 18 × 18 cm^2^, voxel size = 0.6 × 0.6 × 3 mm^3^,
- a diffusion weighted imaging (DWI) sequence with small FOV: TR = 6643 ms, TR = 79.5 ms, FOV = 18 × 18 cm^2^, voxel size = 1.8 × 1.8 × 3 mm^3^; b = 50, 800, 1400; 2000 s/mm^2^ and ADC maps,
- T1-Lava Flex sequence of the whole pelvic region pre-contrast and post-contrast: TR = 5 ms, TE = 1.7 ms, FOV: 44 × 35.2 cm^2^, voxel size = 1.3 × 1.2 × 2 mm^3^,
- a high temporal resolution T1 perfusion sequence after IV injection of 0.1 mmol/kg bolus of gadobutrol (Gadovist, Bayer Schering Pharma, Germany) at a flow rate of 3.5 mL/s: DISCO, TR = 5.1 ms, TE = 1.7 ms, FOV = 29 × 29 cm^2^, Voxel size = 1.9 × 2.2 × 3 mm ^3^, 88 dynamics.

Following the single bed HS acquisition, a total-body (TB) PET scan (5–6 FOVs, 4 min/FOV) was then simultaneously acquired to a MRI TB T1 Lava Flex sequence for anatomical localization, and a TB DWI with b = 50 and b = 1000 s/mm^2^.

PET images were reconstructed using a Bayesian penalized likelihood reconstruction algorithm with a reconstructed FOV of 60 cm and image matrix of 192 × 192. The algorithm includes a Point Spread Function and Time of Flight information.

Attenuation Correction (AC) of PET data was performed using MR-Based AC technique based on the processing of the LAVA-Flex sequences acquired simultaneously with the PET data.

### 2.3. ^68^Ga-DOTA-RM2 PET/MRI Acquisition Protocol

^68^Ga-DOTA-RM2 was synthetized according to the procedure previously described in [12].

^68^Ga-DOTA-RM2 PET/MRI scan was performed within sixteen days (mean: 3 days, range: 2–16 days) from ^68^Ga-PSMA PET/MRI; as for ^68^Ga-PSMA PET/MRI, fasting condition was requested on the day of the examination and the same PET/MRI scanner was used.

Image acquisition started approximatively 50 min (mean ± SD, 49 ± 13 min) after injection of 84–228 MBq (mean ± SD, 153.36 ± 36 MBq) of ^68^Ga-DOTA-RM2 [10,14]. The ^68^Ga-DOTA-RM2 PET/MR examination protocol included a HS scan of the pelvis (20 min), covering a single bed position, that was simultaneously acquired with an axial T2 weighted sequence with large FOV (32 × 32 cm^2^) for anatomical localisation.

Following the single bed acquisition, a TB PET scan (5–6 FOVs, 4 min/FOV) was acquired from the base of the skull to mid-thigh simultaneously with a MRI TB Lava Flex sequence and TB DWI with b = 50 and b = 1500 s/mm^2^.

Reconstruction and attenuation correction of PET images were performed by using the same algorithms and parameters used for ^68^Ga-PSMA PET images.

### 2.4. PET/MR Image Analysis

^68^Ga-PSMA and ^68^Ga-DOTA-RM2 image read-out was performed on the Advantage Workstation (AW, General Electric Healthcare, Waukesha, WI, USA), on which PET, MRI and fused PET/MR images could be visualized in axial, coronal, and sagittal planes. HS PET acquisition bed on the pelvic region and TB PET examination of both ^68^Ga-PSMA and ^68^Ga-DOTA-RM2 PET/MRI images were qualitatively interpreted by two experienced (more than 10 years of experience) Nuclear Medicine physicians, with knowledge of all the available patients’ clinical and imaging information. MR examination being part of the patients’ clinical work-up, MR images were evaluated by a Radiologist Physician and a report was provided to each patient. As the present study is specifically focused on the use of ^68^Ga-PSMA and ^68^Ga-DOTA-RM2 PET in recurrent PCa, MRI data were not considered for the purpose of this work and therefore were not included in the analysis. MRI was just used for the localization of the lesions.

The whole-body distribution pattern of both ^68^Ga-PSMA and ^68^Ga-DOTA-RM2 was qualitatively assessed, and the presence of increased uptake was considered positive for malignancy, with the exception of areas of physiologically increased uptake [18,19]. The anatomical sites were defined based on MR images simultaneously acquired with PET examinations.

Regions of interest (ROI) were semi-automatically defined on transaxial PET images on the single site of recurrence in those patients presenting only one pathological finding and on the most representative site of pathological uptake in those patients presenting multiple lesions. Furthermore, SUVmax and SUVmean, calculated at the 40% threshold of SUVmax [20], were calculated from each ROI drawn on TB PET images, for both radiotracers.

A qualitative comparison between ^68^Ga-PSMA and ^68^Ga-DOTA-RM2 pathological uptakes in the different anatomical sites localized on MR images was performed in order to describe the possible concordances and discrepancies between ^68^Ga-PSMA and ^68^Ga-DOTA-RM2 PET/MR imaging. Specifically, ^68^Ga-PSMA and ^68^Ga-DOTA-RM2 PET/MR findings were defined concordant if the same pathological lesions were detected by both modalities or if both scans resulted negative for the presence of pathological findings. Moreover, ^68^Ga-PSMA and ^68^Ga-DOTA-RM2 findings were described as partially concordant if at least one lesion, but not all pathological findings, was detected by PET examination with both tracers. Finally, when pathological uptake was observed in one PET examination, but not in the other, the exams were considered discordant.

### 2.5. Lesion Validation

All PET findings were validated by using clinical and instrumental follow-up. Specifically, ^68^Ga-PSMA and ^68^Ga-DOTA-RM2 PET/MR imaging findings were considered true positive when at least one of the following criteria was met: (a) histological confirmation on surgically resected specimen; (b) progression (increase in number of pathological ^68^Ga-PSMA uptake sites or increase in uptake intensity) on follow-up ^68^Ga-PSMA PET/CT or PET/MR studies associated with an increase in PSA level; (c) confirmation on conventional imaging either at baseline (including the diagnostic MR exam performed simultaneously to the ^68^Ga-PSMA scan) or during follow-up; (d) disappearance or considerable reduction (i.e., number and intensity) of ^68^Ga-PSMA uptake on follow-up PET/CT or PET/MR scans after local or systemic treatment associated with a decrease in PSA level greater than 50%; (e) a decrease in PSA level greater than 50% after selective irradiation of the site of pathological ^68^Ga-PSMA or ^68^Ga-DOTA-RM2 uptake.

Patients with negative ^68^Ga-PSMA and/or ^68^Ga-DOTA-RM2 PET/MR were considered true negative in the absence of evidence of disease on conventional imaging or ^68^Ga-PSMA PET/CT or PET/MR acquired during the follow-up period (mean follow-up duration for both positive and negative findings: 13.8 months, range: 8.2–17.9 months).

### 2.6. Statistical Analyses

Statistical analyses were performed with R statistical software [21], and Python version 3.7, Python Software Foundation, available at http://www.python.org. (accessed on 30 November 2021). McNemar’s test was used to test for the differences in detection rate of recurrent PCa according to ^68^Ga-PSMA and ^68^Ga-DOTA-RM2 PET/MRI uptake on a patient basis in those patients examined with both radiotracers. Moreover, in the same patients, Wilcoxon-signed rank test was performed to compare the positive findings of ^68^Ga-PSMA and ^68^Ga-DOTA-RM2 PET/MRI on a lesion basis.

In order to study the association between ^68^Ga-PSMA and ^68^Ga-DOTA-RM2 PET/MRI findings and clinical data, the sample was subdivided based on PSA concentration in three groups (PSA ≤ 0.5 ng/mL, 0.5–2 ng/mL, ≥2 ng/mL); based on PSA doubling time (PSA DT) in two groups (PSA DT <6 months and ≥ 6 months); and based on GS of the primary tumour (prior to radical therapy) in two groups (GS ≤ 3 + 4 and ≥4 + 3). Fisher’s exact test was used to correlate clinical and histopathological data with dual-tracer PET positive findings on a patient basis. Furthermore, Receiver Operating Characteristics (ROC) analysis was computed to investigate the predictive value of clinical and histopathological data (PSA, PSA DT, and GS) to predict ^68^Ga-PSMA and ^68^Ga-DOTA-RM2 PET/MRI positivity. Corresponding area under the curve (AUC) with 95% confidence interval (CI) were derived.

The Mann-Whitney U test was used to investigate whether higher levels of PSA, GS, and lower PSA DT were related to a higher number of PET positive lesions. All *p*-values were corrected for multiple testing by false discovery rate and significance was defined below the 0.05 level.

## 3. Results

### 3.1. Patients

Thirty-five men (mean age: 70 years; range: 49–84) with BCR of PCa were enrolled in this prospective study. Mean PSA level at time of scanning was 1.88 ng/mL (range: 0.21–14.4). All patients underwent ^68^Ga PSMA PET/MRI, with 31/35 also undergoing ^68^Ga-DOTA-RM2 PET/MRI because of reduced compliance to the study protocol. Patients’ characteristics are reported in Table 1.

### 3.2. PET/MRI Findings and Comparison between ^68^Ga-PSMA and ^68^Ga-DOTA-RM2

**Figure 1:**
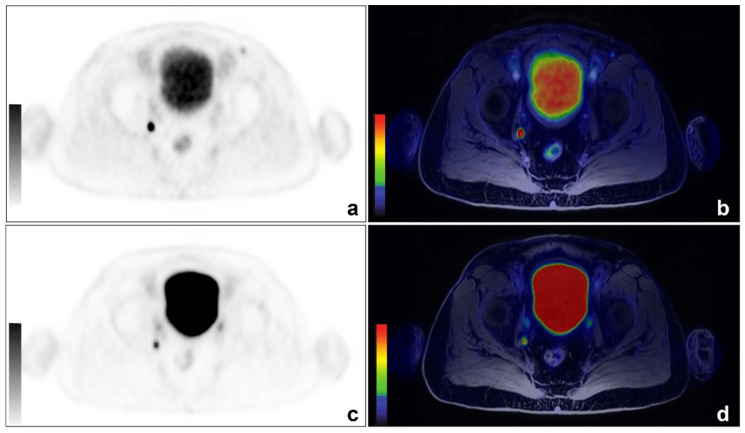
An example of concordant findings between ^68^Ga-PSMA and ^68^Ga-DOTA-RM2 PET/MRI in detecting PCa lymph nodal recurrence. A 66-year-old patient previously treated with radical prostatectomy for a Gleason 7 (4 + 3) PCa (pT3aN0) experienced biochemical recurrence (PSA: 0.53 ng/mL). ^68^Ga-PSMA PET/MRI images showed a focal right obturator lymph nodal uptake ((**a**): axial ^68^Ga-PSMA PET; (**b**): axial ^68^Ga-PSMA PET/MRI). The same finding is visible on ^68^Ga-DOTA-RM2 PET/MRI images (**c**): axial ^68^Ga-DOTA-RM2 PET; (**d**): axial ^68^Ga-DOTA-RM2 PET/MRI).

^68^Ga-PSMA PET/MRI was positive in 26/35 patients (74%), with a total number of 95 lesions being detected (mean SUVmax: 19.69, range: 1.91–112.37; mean SUVmean: 12.03, range: 1.04–65.83); ^68^Ga-DOTA-RM2 PET/MRI was positive in 15/31 patients (48%), with a total number of 41 detected lesions (mean SUVmax: 19.51, range: 3.41–146.62; mean SUVmean: 13.36, range: 2.04–101.41). ^68^Ga-PSMA and ^68^Ga-DOTA-RM2 PET/MRI findings were concordant in 12/31 patients (6 patients with the same positive findings on both examinations, 6 patients with negative examinations; Figure 1 for an exemplar image), partially concordant in 8/31 patients and discordant in 11/31 patients. Specifically, regarding the discordant findings, ^68^Ga-PSMA detected 7 lesions in 5 patients having local recurrences, 5 lesions in 5 patients experiencing bone disease, 4 lesions in 3 patients with lymph nodal recurrence and 2 lesions in one patient with visceral metastases. Conversely, ^68^Ga-DOTA-RM2 PET/MRI detected only one lesion in one patient presenting bone involvement.

Among the 12 concordant cases, 6/12 were confirmed as true positive and 4/12 true negative, whilst 2/12 patients were found to be false negative. Of note, regarding patient n. 9 who resulted to be true positive, further examination on conventional imaging confirmed only a minority of lesions detected by PET with both radiotracers.

Among patients presenting partially concordant uptake findings of the two radiotracers, in only one subject ^68^Ga-DOTA-RM2 detected more lesions compared to ^68^Ga-PSMA; notably, these additional findings were not confirmed by available evidence gathered in the follow-up period. Conversely, in 5/7 patients for whom ^68^Ga-PSMA identified more locations of PCa recurrence than ^68^Ga-DOTA-RM2, the results were confirmed by follow-up examination.

**Figure 2:**
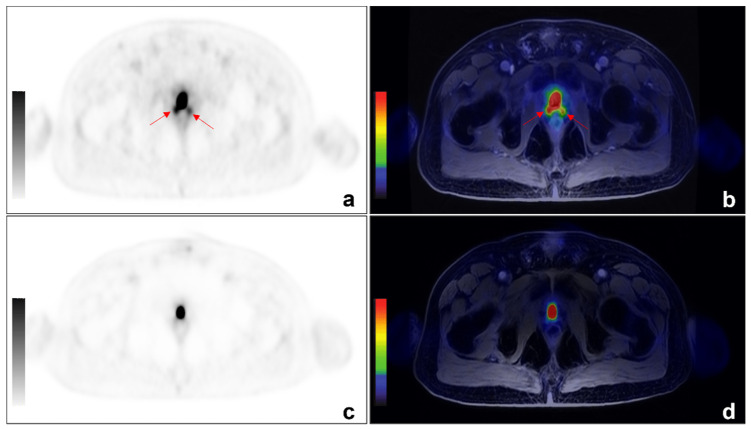
An example of discordant findings between ^68^Ga-PSMA and ^68^-Ga-DOTA-RM2 PET/MRI in detecting PCa local recurrence. A 64 year-old patient previously treated with radical prostatectomy for PCa (Gleason 4 + 3) experienced biochemical recurrence of PCa (PSA = 2.5 ng/mL). ^68^Ga-PSMA PET/MRI showed radiotracer uptake (red arrows) in correspondence of the right and left posterior sides of the prostatic lodge. ((**a**): axial ^68^Ga-PSMA PET; (**b**): axial ^68^Ga-PSMA PET/MRI). No pathological uptake was detected on ^68^Ga-DOTA-RM2 PET/MR images (**c**): axial ^68^Ga-DOTA-RM2 PET; (**d**): axial ^68^Ga-DOTA-RM2 PET/MRI).

**Figure 3:**
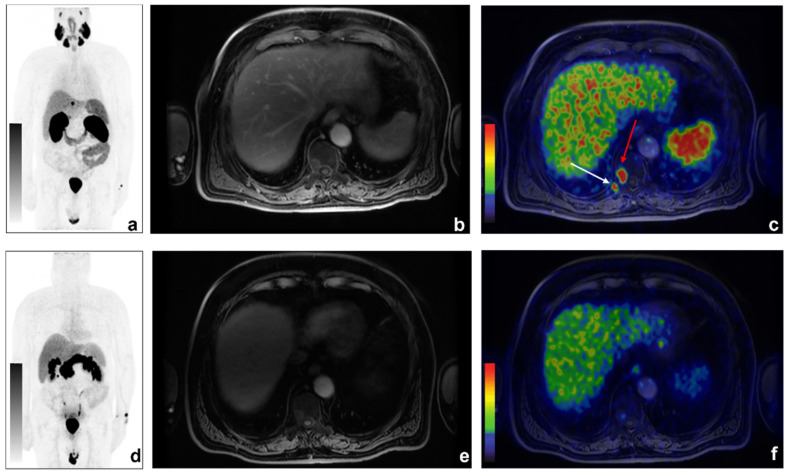
An example of discordant findings between ^68^Ga-PSMA and ^68^Ga-DOTA-RM2 PET/MRI in bone metastases detection. A 70-year-old patient previously treated with radical prostatectomy for PCa (Gleason 5 + 4; pT3aN0) experienced biochemical recurrence of PCa (PSA = 2.16 ng/mL). ^68^Ga-PSMA PET/MRI showed radiotracer uptake in correspondence of right D10 hemisome ((**a**): ^68^Ga-PSMA MIP; (**b**): axial LAVA-FLEX MRI; (**c**): axial ^68^Ga-PSMA PET/MRI, red arrow) and the right posterior tract of the X rib (**c**): white arrow). Non-significant uptake was detected on ^68^Ga-DOTA-RM2 PET/MR images (**d**): ^68^Ga-DOTA-RM2 MIP; (**e**): axial LAVA-FLEX MRI; (**f**): ^68^Ga-DOTA-RM2 PET/MRI).

Among the 11/31 discordant findings, 10/11 were ^68^Ga-PSMA positive and ^68^Ga-DOTA-RM2 negative (Figure 2 and Figure 3 for exemplar images).

Nine/11 of these patients were confirmed to be true positive by follow-up examination, while 1/11 ^68^Ga-PSMA positive finding was not supported by further evidence, thus being classified as false positive. The remaining patient presenting ^68^Ga-DOTA-RM2 positive finding with negative ^68^Ga-PSMA PET/MRI, finally resulted to be a true positive. See Table 2 for detailed information on the follow-up examination for each patient.

**Figure 4:**
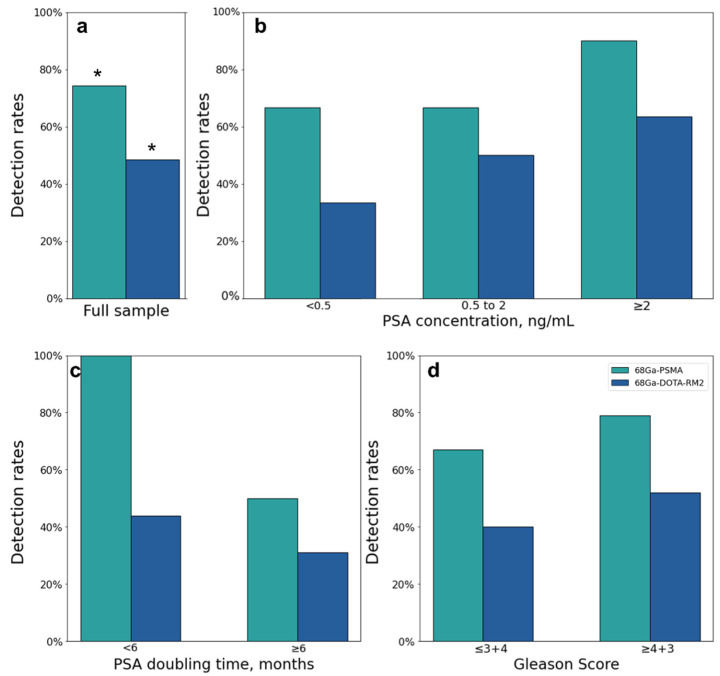
^68^Ga-PSMA and ^68^Ga-DOTA-RM2 PET/MRI findings. (**a**): ^68^Ga-PSMA and ^68^Ga-DOTA-RM2 detection rates; (**b**): ^68^Ga-PSMA and ^68^Ga-DOTA-RM2 detection rates stratified by different PSA levels at time of scans; (**c**): ^68^Ga-PSMA and ^68^Ga-DOTA-RM2 detection rates stratified by PSA DT; (**d**): ^68^Ga-PSMA and ^68^Ga-DOTA-RM2 detection rates stratified by GS prior to radical treatment. * = *p* < 0.05.

**Figure 5:**
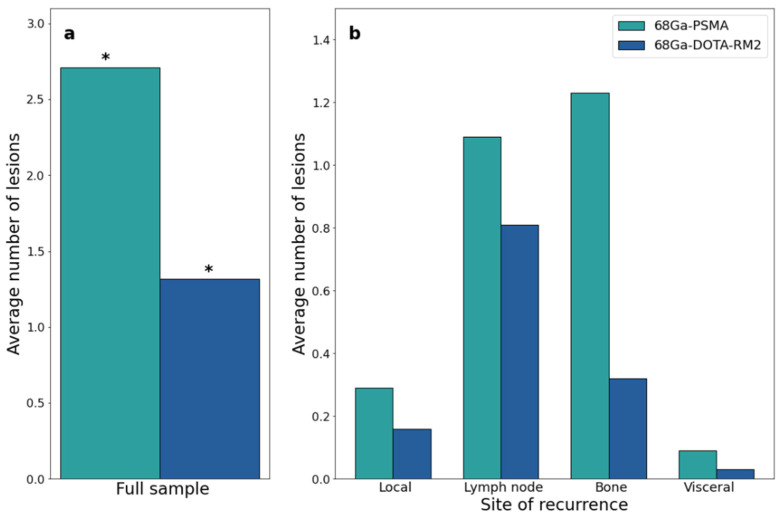
^68^Ga-PSMA and ^68^Ga-DOTA-RM2 PET/MRI findings at the lesion level. (**a**): Average number of ^68^Ga-PSMA and ^68^Ga-DOTA-RM2 PET/MRI positive lesions; (**b**): Average number of ^68^Ga-PSMA and ^68^Ga-DOTA-RM2 PET/MRI positive lesions stratified by site. * = *p* < 0.05.

Both scans identified 26 lesions in 19/31 patients, while ^68^Ga-PSMA detected 66 additional lesions in 17/31 patients as compared to ^68^Ga-DOTA-RM2. Fifteen lesions in 3/31 patients were visible only on ^68^Ga-DOTA-RM2 PET/MRI. The patient-based detection rates (Figure 4a) and the average number of detected lesions (Figure 5a) were significantly different between the two imaging modalities (*p* = 0.016 and 0.002). The detection rates of these radiotracers stratified by PSA serum concentration, PSA DT and GS prior to radical treatment are depicted in Figure 4b, Figure 4c and Figure 4d, respectively. Moreover, in Figure 5b is shown the average number of lesions identified by dual-tracer PET stratified by site of recurrence.

### 3.3. Associations between Semi-Quantitative Imaging Parameters and Clinical Data

Correlations between clinical data and ^68^Ga-PSMA and ^68^Ga-DOTA-RM2 detection rates on a patient basis are reported in Table 3. PSA level and GS were not significantly associated with ^68^Ga-PSMA PET/MRI detection rate, while patients with higher PSA DT, were less likely to be ^68^Ga-PSMA PET positive compared to those showing PSA DT < 6 months (OR < 0.01, *p* = 0.022). However, this association was lost after correction for multiple testing (adjusted *p* = 0.065). None of the investigated clinical variables was significantly associated with ^68^Ga-DOTA-RM2 PET/MRI detection rate on a patient basis.

**Figure 6:**
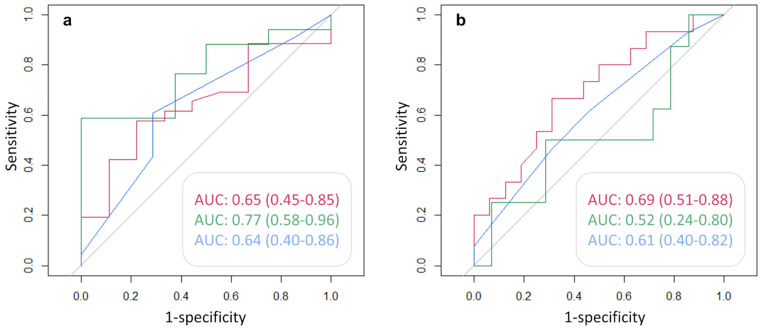
ROC analysis for the prediction of (**a**): ^68^Ga-PSMA and (**b**): ^68^Ga-DOTA-RM2 PET positive findings. Red line: AUC of PSA at time of scans in the prediction of PET positivity; green line: AUC of PSA DT in the prediction of PET positivity; blue line: AUC of GS prior to radical treatment in the prediction of PET positivity. AUC and 95% CI are here reported.

Figure 6 shows the AUC, and the corresponding 95% CI, of each clinical variable used to predict the positivity of either ^68^Ga-PSMA (Figure 6a) or ^68^Ga-DOTA-RM2 (Figure 6b) PET/MRI in the detection of recurrent PCa. PSA DT has an AUC = 0.77 (95% CI = 0.58, 0.96) for the prediction of positive ^68^Ga-PSMA PET, while PSA at time of scans presents an AUC = 0.69 for the prediction of ^68^Ga-DOTA-RM2 positive findings, although the 95% CI touches the diagonal (random classification). All the other investigated variables do not bear the potential to predict neither ^68^Ga-PSMA nor ^68^Ga-DOTA-RM2 PET positivity for the identification of recurrent PCa in our cohort.

Table 4 reports the number of lesions detected by PET/MRI stratified according to GS, PSA levels and kinetic.

**Figure 7:**
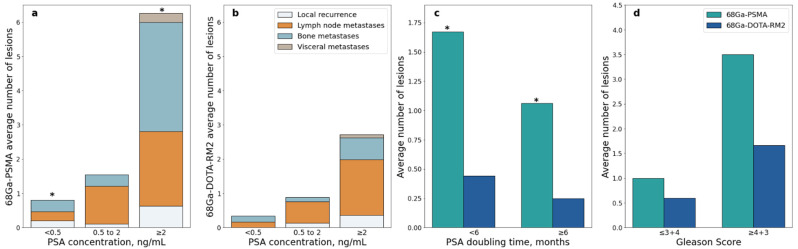
^68^Ga-PSMA and ^68^Ga-DOTA-RM2 PET/MRI detection rates stratified according to clinical data; (**a**): Average number of ^68^Ga-PSMA PET/MRI positive lesions stratified by site and PSA level at time of scans; (**b**): Average number of ^68^Ga-DOTA-RM2 PET/MRI positive lesions stratified by site and PSA level at time of scans; (**c**): Average number of ^68^Ga-PSMA and ^68^Ga-DOTA-RM2 PET/MRI positive lesions stratified by PSA doubling time; (**d**): Average number of ^68^Ga-PSMA and ^68^Ga-DOTA-RM2 PET/MRI positive lesions stratified by Gleason Score, * = *p* < 0.05.

A significant difference in the number of lesions detected by ^68^Ga-PSMA PET/MRI was observed in patients with PSA value < 0.5 ng/mL vs. PSA value ≥ 2 ng/mL (Mann-Whitney U = 21.5, *p* = 0.001, Figure 7a); this difference remained significant after correction for multiple testing (adjusted *p* = 0.006). A significant difference was also observed in patients with PSA DT < 6 months vs. PSA DT ≥ 6 months (Mann-Whitney U = 119, adjusted *p* = 0.044; Figure 7c).

Similarly, higher PSA serum levels were associated with more PET positive lesions in ^68^Ga-DOTA-RM2 PET/MRI, but significance was lost after correction for multiple testing (Mann-Whitney U = 46, adjusted *p* = 0.17; Figure 7b). The number of PET positive lesions did not differ significantly across GS levels both on ^68^Ga-PSMA and ^68^Ga-DOTA-RM2 PET/MRI (Mann-Whitney U = 56, adjusted *p* = 0.43 and U = 66.5, adjusted *p* = 0.65, respectively; Figure 7d).

## 4. Discussion

The present study investigated the diagnostic performance of ^68^Ga-PSMA and ^68^Ga-DOTA-RM2 PET/MRI for detection of recurrent PCa after primary treatment. Furthermore, we also studied the association between ^68^Ga-PSMA and ^68^Ga-DOTA-RM2 PET uptake and clinical as well as histopathological data.

So far, only few studies have investigated the combined use of both ^68^Ga-PSMA and ^68^Ga-DOTA-RM2 PET in PCa both in the staging and restaging setting, using PET/MRI and PET/CT alternatively [10,12,13,14].

Recently, Baratto et al. conducted the largest study to date investigating the use of ^68^Ga-PSMA and ^68^Ga-DOTA-RM2 in patients with recurrent PCa [10]. However, despite the lower number of patients included in the analysis, our study presents several methodological advantages. In fact, in the present clinical trial, all patients have been examined with both ^68^Ga-PSMA and ^68^Ga-DOTA-RM2 PET/MRI, thus not alternatively using either ^68^Ga-PSMA or ^18^F-PSMA as previous groups have done; patients’ recruitment and data analysis have been prospectively performed and all findings were validated by clinical and/or instrumental follow-up.

In our cohort, ^68^Ga-PSMA identified PCa recurrent lesions in 74% of patients, while ^68^Ga-DOTA-RM2 PET/MRI was positive in only 48% of patients. Interestingly, 9/10 of the patients presenting ^68^Ga-PSMA, but not ^68^Ga-DOTA RM2 uptake were confirmed to be true positive by follow-up examination. This is in contrast with previous findings where PSMA and ^68^Ga-DOTA-RM2 PET had similar detection rates on a patient basis [10].

On a lesion-based analysis, our results are coherent with the ones reported by Baratto et al., which directly compared the performances of ^68^Ga-PSMA and ^68^Ga-DOTA-RM2 PET and found that PSMA detected 36 additional lesions in 13 patients, with 66 additional lesions in 17 patients observed in our study.

The ability of PET to detect BCR at low PSA levels allows a personalized treatment planning at an early stage of recurrent disease by permitting early local therapies with curative intent. Therefore, it is particularly relevant to investigate the performance of different PET tracers by stratifying their detection rate according to PSA concentration. For this purpose, most studies divided their sample according to PSA in 5 sub-groups (<0.5, 0.5–1, 1–2, 2–5, >5) [10,22,23]. Differently from previous published data, in the present work, in order to allow statistical analysis, the cohort was stratified according to PSA level in 3 groups (<0.5, 0.5–2, >2) because of the numerosity of the cohort. The missing association between ^68^Ga-PSMA PET/MRI positivity rate and PSA level found in our cohort is likely due to the limited numerosity of the investigated sample combined with the high detection rate of ^68^Ga-PSMA for low PSA values (66.7% for PSA < 0.5 ng/mL). These findings are in line with previous works assessing the performance of PSMA in detecting recurrent PCa in larger cohorts [24,25,26], but higher compared to the detection rates reported in similar settings [10,22,27,28].

However, similarly to previous studies [22,27], we observed that patients presenting PSA concentration ≥2 ng/mL at the time of scans showed a significantly higher number of ^68^Ga-PSMA PET/MRI positive lesions compared to patients with PSA level <0.5 ng/mL.

Although the association between ^68^Ga-DOTA-RM2 PET/MRI positivity rate and PSA concentration both on a patient and lesion basis was not significant, we observed a trend towards a higher rate of ^68^Ga-DOTA-RM2 PET/MRI positive findings at higher PSA values, confirmed also by ROC analysis, that will surely be an interesting matter of study in future investigations.

The link between PSA kinetics and ^68^Ga-PSMA PET findings is well established, with patients having slow PSA kinetics being less likely to be ^68^Ga-PSMA PET positive [29,30]. Accordingly, we reported that patients having a PSA DT ≥ 6 months present a significantly lower number of lesions compared to those having a faster rise in PSA levels after primary treatment; we observed the same trend on a patient-based analysis, although after adjustment for multiple testing, the *p*-value only approached the level of significance (*p* = 0.065). Furthermore, ROC analysis showed that PSA DT has a moderate predictive value in the identification of ^68^Ga-PSMA PET positivity, in line with the correlation previously investigated. Conversely, we did not detect any association between ^68^Ga-DOTA-RM2 PET/MRI positivity rate and PSA kinetics.

Currently, the possible association between GS of the primary tumour and ^68^Ga-PSMA PET positive findings in recurrent PCa has not been definitively demonstrated, with different studies reporting conflicting results [10,24]. In our study, we did not observe any association between the positivity rate of PET/MRI with either radiotracer and GS.

A constraint of the present study might be the relatively limited number of included patients. However, as mentioned above, our cohort is remarkably homogeneous as all patients have been examined on the same scanner and applying the same methodology (all PET/MRI studies and not both PET/MRI and PET/CT; exclusive use of ^68^Ga-PSMA and ^68^Ga-DOTA-RM2).

Another limitation might be the lack of post-hoc correlation of imaging findings with histopathological results for all patients; however, we consider this a relative limitation related to patients’ disease management, as a number of findings detected on PET/MRI are located in sites that are not usually considered for bioptic confirmation (i.e., bone) and/or are directly treated without any further confirmation (i.e., radiotherapy on positive lymph-nodes, ADT). Additionally, all PET findings were validated using clinical and instrumental follow-up. All images were acquired using the same Signa 3 Tesla PET/MRI scanner, but since this study is part of a single-centre prospective trial that is still ongoing at IRCCS San Raffaele Scientific Institute, and it was specifically focused on the use of ^68^Ga-PSMA and ^68^Ga-DOTA-RM2 PET in recurrent PCa, MRI data were not considered for the purpose of this work and therefore were not included in the analysis. The inclusion of MR data will surely constitute an improvement of the present work and will be a matter of investigation in future studies.

Finally, the two PET examinations were read separately, with the Nuclear Medicine physicians not being blind to the clinical history of the patients, thus resulting in a potential bias.

## 5. Conclusions

The possibility to identify different sites of recurrence of PCa after radical treatment by using a dual-tracer approach certainly improves the disease characterization and therefore it may ultimately have an impact on patients’ management and follow-up. Although in our cohort, ^68^Ga-PSMA had a higher detection rate than ^68^Ga-DOTA-RM2 in detecting recurrent PCa. Furthermore, the number of ^68^Ga-PSMA PET/MRI positive lesions was associated with higher levels of PSA and a shorter PSA DT, thus representing potential prognostic factors. GS, on the other hand, was not related to PET findings.

## Figures and Tables

**Table 1:** Patients’ characteristics.

Pt	Age (years)	GS	PSA (ng/mL)	Treatment	Adjuvant Therapy
1	64	4 + 4	9.86	RP	None
2	66	4 + 3	0.53	RP	None
3	75	3 + 3	0.54	RP	None
4	78	4 + 5	0.212	RP	RT
5	73	4 + 3	4.75	RP	RT
6	77	4 + 4	0.237	RP	None
7	58	4 + 3	1.3	RP	None
8	64	4 + 3	2.5	RP	RT
9	79	4 + 5	2	RP	RT; ADT
10	49	4 + 5	0.82	RP	None
11	84	NA	3.24	RT	None
12	79	5 + 3	1.53	RP	RT; ADT
13	75	3 + 4	0.39	RP	None
14	78	4 + 5	0.5	RP	RT
15	76	4 + 5	0.41	RP	RT
16	67	4 + 4	2	RP	None
17	76	3 + 4	0.41	RP	None
18	81	NA	3.53	RT	None
19	71	4 + 3	4.3	RP	RT
20	72	3 + 4	0.22	RP	RT
21	77	2 + 4	1.21	RP	None
22	77	4 + 5	4.63	RP	RT
23	50	5 + 5	14.4	RP	None
24	69	4 + 5	0.2	RP	None
25	59	4 + 4	0.26	RP	None
26	53	NA	0.25	RP	None
27	75	4 + 3	0.27	RP	None
28	62	3 + 3	0.68	RP	None
29	70	5 + 4	2.16	RP	None
30	73	4 + 3	0.4	RP	None
31	64	4 + 5	0.23	RP	None
32	69	NA	0.37	RP	RT
33	74	5 + 4	0.74	RP	RT
34	62	5 + 4	0.34	RP	RT
35	60	4 + 3	0.48	RP	None

RP: radical prostatectomy; RT: radiotherapy; LN: lymph-node; ADT: androgen deprivation therapy; PSA at time of scans; NA: not available.

**Table 2:** PET findings and PET findings validation.

Pt	^68^Ga-PSMA Findings	^68^Ga-DOTA-RM2 Findings	PET Findings Validation
1	Left perirectal lesion	Left perirectal lesion	Confirmation on conventional imaging at baseline
2	Right obturator LN; right laterocervical LN	Right obturator LN	Right obturator LN confirmed on conventional imaging at baseline
3	Negative	Negative	No evidence of disease on conventional imaging either at baseline or follow-up
4	Left humerus	NA	Decrease in PSA level greater than 50% after RT on the site of pathological ^68^Ga-PSMA uptake
5	Left supraclavicular LN; 2 left paraortic LNs; left iliac bone; left sacral ala	Left synchondrosis; 8 left paraortic LNs; interaortocaval LN; left retroclavicular LN; 2 right retrocrural LNs; 2 left common iliac LNs	Left iliac bone and left sacral ala confirmed on conventional imaging at baseline
6	Negative	NA	No evidence of disease on conventional imaging either at baseline or follow-up and stable level of PSA during follow-up
7	Negative	Negative	No evidence of disease on conventional imaging or ^11^C-choline and ^68^Ga-PSMA PET either at baseline or follow-up
8	Bilateral prostatic fossa (2)	Negative	Progression on follow-up ^68^Ga-PSMA PET studies associated with an increase in PSA level
9	Left lateral rectal wall; left common iliac LN; left paramedian presacral LN	left lateral rectal wall; left common iliac LN; left paramedian presacral LN	Left lateral rectal wall confirmed on conventional imaging at baseline
10	Right iliac ala	NA	Decrease in PSA level greater than 50% after RT on the site of pathological ^68^Ga-PSMA uptake
11	Right prostate lobe; right internal iliac LN; left iliac bone	Right prostate lobe	Disappearance of ^68^Ga-PSMA uptake on follow-up PET scans after systemic treatment associated with a decrease in PSA level greater than 50%
12	Right vesical-urethral anastomosis; 2 left laterocervical LNs; left retroclavicular LN; left dorsal LN	Right vesical-urethral anastomosis	Right vesical-urethral anastomosis confirmed on conventional imaging at baseline
13	Right vesical-urethral anastomosis; left common iliac LN	Left common iliac LN	Confirmation of ^68^Ga-PSMA PET findings on conventional imaging at baseline
14	Negative	Negative	Evidence of disease on conventional imaging at baseline (vesical-urethral anastomosis and right iliac bone) and increase in PSA level during follow-up
15	Right vesical-urethral anastomosis	Negative	Confirmation of ^68^Ga-PSMA PET findings on conventional imaging at baseline
16	Left pubis; right V rib	Negative	Left pubis confirmed by conventional imaging at baseline
17	Negative	NA	Evidence of disease on conventional imaging at baseline (vesical-urethral anastomosis)
18	Negative	Negative	No evidence of disease on conventional imaging either at baseline or follow-up
19	Left pulmonary hilum; left acetabulum	Left pulmonary hilum	Disappearance of ^68^Ga-PSMA uptake on follow-up PET scans after systemic treatment associated with a decrease in PSA level greater than 50%
20	Left retrolateral vesical-urethral anastomosis	Negative	Confirmation of ^68^Ga-PSMA PET findings on conventional imaging at baseline
21	Left obturator LN, left III rib	Left obturator LN; left III rib	Confirmed on conventional imaging at baseline
22	Bilateral iliac LNs (2); left rectus abdominis muscle; bilateral pleura (2)	Negative	Confirmation of ^68^Ga-PSMA PET findings on conventional imaging at baseline. Pleura confirmed on histological analysis of surgically resected specimens.
23	Multiple LNs (16); multiple skeletal lesions (27)	Left retroclavicular LN; left paraortic LN; multiple hips (6)	Multiple LN and skeletal lesions confirmed on conventional imaging at baseline
24	Right perirectal LN; right pubic bone	Negative	Confirmation of ^68^Ga-PSMA PET findings on conventional imaging at baseline
25	Small trochanter	Negative	Confirmation of ^68^Ga-PSMA PET findings on conventional imaging at baseline and decrease in PSA level greater than 50% after RT on the site of pathological ^68^Ga-PSMA uptake
26	Negative	Negative	Evidence of disease on conventional imaging at baseline (paraortic and aortocaval LN)
27	Right VIII rib	Negative	No evidence of disease on conventional imaging either at baseline or follow-up
28	D7 right hemisome	Negative	Confirmation of ^68^Ga-PSMA PET findings on conventional imaging at baseline
29	D10 right hemisome, right X rib, D8	D10 right hemisome	Confirmation of ^68^Ga-PSMA PET findings on conventional imaging at baseline
30	Negative	Negative	No evidence of disease on conventional imaging either at baseline or follow-up
31	Negative	Right thigh-bone	Confirmation of ^68^Ga-DOTA-RM2 PET findings on conventional imaging at baseline
32	Paracaval LN	Negative	Confirmation of ^68^Ga-PSMA PET findings on conventional imaging at baseline
33	Right iliac LN; 2 right obturator LNs	Right iliac LN; 2 right obturator LNs	Confirmed on conventional imaging at baseline and disappearance of ^68^Ga-PSMA uptake on follow-up PET scans after systemic treatment associated with a decrease in PSA level greater than 50%
34	Left IX rib	Left IX rib	Confirmed on conventional imaging at baseline
35	Right obturator LN	Right obturator LN	Confirmed on conventional imaging at baseline

LN: lymph node; NA: not available.

**Table 3:** Correlations between ^68^Ga-PSMA and ^68^Ga-DOTA-RM2 detection rates and clinical data on a patient basis.

Imaging Modality	Stratification	No. of Patients	Positive Results, No. (%)	*p* Value	Adjusted *p* Value
^68^Ga-PSMA PET/MRI	PSA				
	<0.5	15	10 (67)	0.339	0.509
	0.5–2	9	6 (67)		
	≥2	11	10 (91)		
	PSA DT				
	<6	9	9 (100)	0.022	0.065
	≥6	16	8 (50)		
	Not available	10	9 (90)		
	GS				
	≤3 + 4	6	4 (67)	0.603	0.603
	≥4 + 3	24	19 (79)		
	Not available	5	3 (60)		
^68^Ga-DOTA-RM2 PET/MRI	PSA				
	<0.5	12	4 (33)	0.390	0.993
	0.5–2	8	4 (50)		
	≥2	11	7 (64)		
	PSA DT				
	<6	9	4 (44)	0.662	0.993
	≥6	13	4 (31)		
	Not available	9	7 (78)		
	GS				
	≤3 + 4	5	2 (40)	1	1
	≥4 + 3	21	11 (52)		
	Not available	5	2 (40)		

PSA: Prostate Specific Antigen (ng/mL); PSA DT: PSA Doubling Time (months); GS: Gleason Score.

**Table 4:** Study of the differences in the number of lesions detected by ^68^Ga-PSMA and ^68^Ga-DOTA-RM2 and clinical data.

Imaging Modality	Stratification	Positive Lesions, No. (Average)
^68^Ga-PSMA PET/MRI	PSA	
	<0.5	12 (0.8)
	0.5–2	14 (1.56)
	≥2	69 (6.27)
	PSA DT	
	<6	15 (1.67)
	≥6	17 (1.06)
	Not available	63 (6.3)
	GS	
	≤3 + 4	6 (1)
	≥4 + 3	84 (3.5)
	Not available	5 (1)
^68^Ga-DOTA-RM2 PET/MRI	PSA	
	<0.5	4 (0.33)
	0.5–2	7 (0.88)
	≥2	30 (2.73)
	PSA DT	
	<6	4 (0.44)
	≥6	4 (0.31)
	Not available	33 (3.66)
	GS	
	≤3 + 4	3 (0.6)
	≥4 + 3	36 (1.71)
	Not available	2 (0.4)

PSA: Prostate Specific Antigen (ng/mL); PSA DT: PSA Doubling Time (months); GS: Gleason Score.

## Data Availability

All code needed to replicate our analyses is available upon request from the corresponding author.
